# Trichoblastoma mimicking basal cell carcinoma and the approach to its management: Case report

**DOI:** 10.1016/j.ijscr.2021.106318

**Published:** 2021-08-18

**Authors:** Nadeen Al Mushcab, Raja Husain, Mohammad Al Subaiei, Ayedh Al Qarni, Ahmed Abbas, Mohammad Al Duhileb

**Affiliations:** a Breast and Endocrine Surgery Department, King Fahad Specialist Hospital, Dammam, Saudi Arabia; b Department of Pathology, King Fahad Specialist Hospital, Dammam, Saudi Arabia

**Keywords:** Case report, Trichoblastoma, Basal cell carcinoma, Diagnosis, Treatment

## Abstract

**Introduction and importance:**

Trichoblastoma is a benign and rare cutaneous lesion with striking resemblance to basal cell carcinoma. However, the course of the illness varies significantly from basal cell carcinoma. It usually takes a less malicious route and causes minimal harm to the patient,unlike basal cell carcinoma where it can be aggressive and requires additional radiotherapy. Therefore, being able to differentiate them from one another is crucial to properly set a management plan.

**Case presentation:**

A 44-year-old Saudi male, diagnosed with basal cell carcinoma of the scalp in Dammam Medical Centre (DMC) and was referred to our hospital for re-excision of positive margins. Slide review of the histopathology samples in our hospital showed features consistent with trichoblastoma. This was further confirmed by a dermahistopathologist.

**Clinical discussion:**

This rare benign tumour has posed multiple challenges in the clinical setting as not enough studies were conducted in order to dictate a proper management course. However, current management approach to the majority of skin lesions rely on the histopathological subtype and surgical excision. Many benign and malignant dermatological entities may mimic basal cell carcinoma, and therefore misdiagnosis can lead to either unnecessary excision or delayed treatment of metastatic disease. It is important to request the proper investigations to ease the process of diagnosis and more importantly, the process of differentiation.

**Conclusion:**

Differentiating the two tumours from one another allows for the initiation of proper treatment early resulting in an improved prognosis for both lesions. The gold standard investigation to diagnose and differentiate between them is a surgical biopsy. Trichoblastoma typically has a favourable prognosis with a low incidence of recurrence or progression.

## Highlights

• Trichoblastoma is rare but its importance lies in its resemblance with basal cell carcinoma.
• The ability to differentiate between trichoblastoma and basal cell carcinoma is crucial as their management differs.
• Proper investigative measures need to be undertaken to ensure proper diagnosis of trichoblastoma.
• A case report depicting management challenges in trichoblastoma

## 1 Introduction and importance

Trichoblastoma is a rare benign cutaneous lesion characterized by the presence of follicular germinative (basaloid) cells [1]. The usual clinical appearance of trichoblastoma is a skin-coloured, slow-growing papule or nodule, typically located on the face or scalp. One of the main differentials for trichoblastoma to consider is basal cell carcinoma (BCC) since the two hold overlapping features both clinically and dermoscopically [2]. It is important to differentiate between the two as one is typically benign and the other usually exhibits malignant behavior. Thus, the management and prognosis for both differs greatly. Here we present a case of trichoblastoma of the scalp and discuss morphological features and management differences between the two tumours.

## 2 Case presentation

A 44-year-old Saudi male, was referred to our clinic from Dammam Medical Centre (DMC) for a scalp lesion suspicious for basal cell carcinoma. The patient initially noticed a mass on his scalp that appeared one year ago, gradually increasing in size. Not associated with pain, discharge, headache or itching. Patient denied any systemic features such as, fever, abdominal pain, nausea or vomiting. He presented to DMC for evaluation as the size was starting to bother him. On examination, there was a scalp lesion 1x2cm in size, regular borders, no signs of inflammation or infection. The patient's past medical history is significant for hypertension and diabetes mellitus. Past surgical history involved a hemorrhoidectomy ten years ago. The patient is on antihypertensive and anti-diabetic medications with no known allergies. No significant family history was noted. The patient subsequently underwent excision of the lesion and subcutaneous tissue under local anaesthesia in DMC. He was discharged on ibuprofen 400 BID PO for five days and omeprazole 40 mg OD PO for seven days. Ten days later, when he represented to the clinic for suture removal, there was a small area of wound dehiscence in the upper edge of the wound. The histopathology report at the time was consistent with basal cell carcinoma, nodular type with deep surgical margins involved by tumour cells. The patient was then referred to our surgical oncology clinic in King Fahad Specialist Hospital (KFSH) for re-excision of positive margins and further management. A slide review (Table 1) was done in our hospital which showed skin adnexal tumour with trichilemmal differentiation favouring trichoblastoma with small tiny foci of tumour cells present in the inferior margin (1 mm). The slide was sent to an expert dermatohistopathologist for a final report which confirmed our findings and a final diagnosis of Trichoblastoma with benign features was made. A computed tomography (CT) scan was done of the head and neck which showed subcentimetric lymph nodes with suspicious morphology involving the posterior aspect of the neck, the post auricular and sub-occipital region. The patient underwent wide local excision under general anaesthesia which was performed by a breast and endocrine surgery consultant. The patient was then discharged home on analgesia and followed up in clinic 10 days later for removal of superficial sutures and alternating removal of deep sutures. He was seen again seven days later for the removal of the remaining sutures.

**Table 1:**
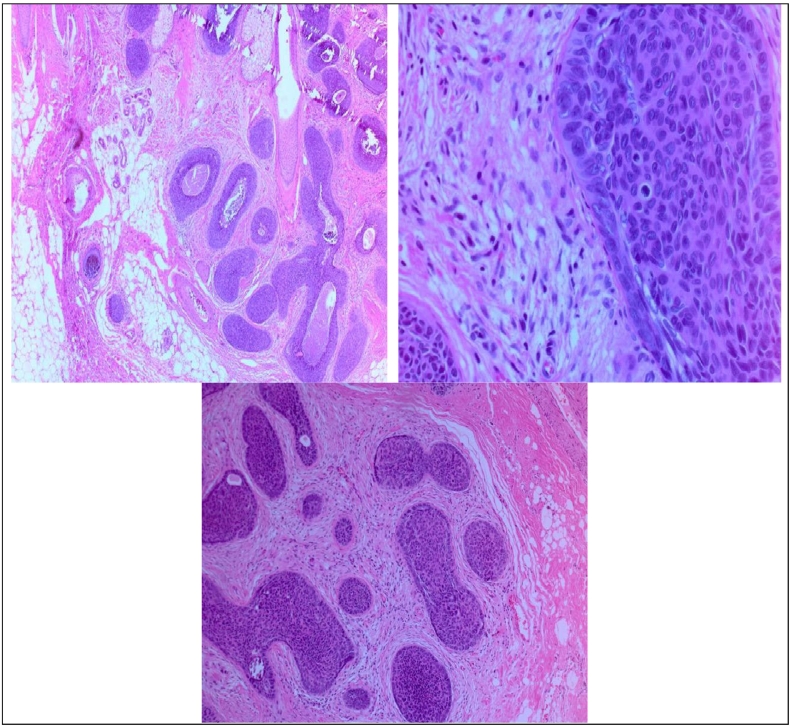
Trichoblastoma showing a dermally centered tumour composed of nests lined by basaloid cells with focal periphera0nfl palisading and stroma. Mitosis and apoptosis are evident. However, cellular pleomorphism is minimal. No clefts, increased dermal mucin, or definite connection to the epidermis is noted. Focal calcification and trichilemmal-type mesenchyme is noted.

## 3 Clinical discussion

Trichoblastoma (TB) is an uncommon skin neoplasm originating in follicular germinative cells. The clinical diagnosis is arduous to make as it is not distinguishable by specific clinical data. It usually presents as an isolated lesion as was the case with our patient. However, it is worth noting that it can also present as multiple lesions [3]. Given the fact that our patient was initially managed outside our hospital, we could not provide any photos of the initial lesion as none were taken.

Basal cell carcinoma (BCC) on the other hand, is the most common malignant cutaneous neoplasm, originating in hair follicle-derived cells. It is known to be polymorphic in clinical appearance ranging from an erythematous plaque to an exophytic tumour with ulcerations [4].

Both cutaneous lesions consist of basaloid cells making their histological diagnosis all the more challenging as a result of the similarities they hold. Due to the varying nature of their treatment, it is prudent to accurately achieve the correct diagnosis in order to precisely dictate the patient's management.

Histologically, TB rarely presents with necrosis, mitotic activity or lymphocytic infiltration. Whereas these are all possible features seen in BCC. TB is generally well demarcated by copious amounts of stromal cells, while BCC's mesenchymal surrounding is less structured and not as abundant. Lastly, neoplastic cells form irregular trabeculae and arrange themselves in palisades at the periphery, which is what is typically seen in TB. In BCC, optically empty clefts are present, referred to as artefact and is usually due to routine tissue processing [5]. The slides were examined by two different pathologists and an expert dermatohistopatholigst and the latter two confirmed TB. This proves clinical experience also plays a bigger role than anticipated in identifying certain features.

Dermoscopically, one of the striking differences seen between TB and BCC was the presence of blue-grey ovoid nests and blue-grey globules. This feature is highly suggestive but not limited to BCC [5]. This was a limitation in our case as the lesion was not investigated dermoscopically.

Numerous studies observed the pattern of cytokeratins (CK5/6, CK14, CK19) as well as the expression of CD34, p53, proliferating cell nuclear antigen (PNCA) and Ki-67. These proteins were found to be expressed in a similar manner in both tumours which could be attributed to the histological origin of the two lesions [6], [7]. Therefore, they provide little diagnostic value to the patients complete work-up. A study conducted by Cordoba et al. looked at the expression of Bcl-2 and CD-10 to support or exclude the diagnosis of TB. It was reported that Bcl-2 was found to be expressed more in the outer most basaloid cells in TB and diffuse in BCC. The central loss of Bcl-2 expression in TB was speculated to be a result of follicular maturation and differentiation. Thus, the use of Bcl-2 is limited to lesions with follicular differentiation. In contrast to CD-10, where the epithelial expression was found to be valuable in detecting areas of proliferation that could be misinterpreted as TB. This finding is paramount to the reaching an accurate diagnosis in patients who will benefit from more suitable treatments [8]. In combination with a histological diagnosis, final needle aspiration biopsy (FNAB) prior to an excisional biopsy was found to be a useful adjunct in aiding the specific diagnosis of TB [9].

Due to the benign nature of TB, it is somewhat challenging for the clinician to decide which lesions warrant excision. The age of the patient and the location should be taken into consideration. However, these benign tumours are unlikely to regress spontaneously [1]. Our patient underwent the aforementioned management option and was followed up twice post-operatively with no further management required.

In comparison to the management of BCC which is far more extensive than TB. The Journal of the American Academy of Dermatology has set proper guidelines of care for the management of BCC with the first line management being surgical resection. If surgical intervention was not feasible, alternative therapies include cryosurgery, topical therapy such as, imiquimod, radiotherapy or laser therapy [10]. The details of these therapies is beyond the scope of this report.

## 4 Conclusion

Histological diagnosis with proper staining is crucial in the differentiation process of trichoblastoma. When physicians suspect basal cell carcinoma, trichoblastoma should be in their list of differentials. and proper workup should be ordered. However, more studies are needed before we can reach a definitive method to establish the diagnosis of trichoblastoma. This case report should serve as an addition to the literature to help further the diagnosis and management of trichoblastoma.

## Scare checklist statement

The manuscript was prepared and revised according to the SCARE guidelines (2020) [11].

## Provenance and peer review

Not commissioned, externally peer-reviewed.

## Sources of funding

N/A.

## Ethical approval

Exempted.

## Consent

Written informed consent was obtained from the patient for publication of this case report and accompanying images. A copy of the written consent is available for review by the Editor-in-Chief of this journal on request.

## Research registration

N/A.

## Guarantor

Dr.Nadeen AlMushcab, Dr.Mohammad Al Duhileb

## Declaration of competing interest

N/A.
